# Ecological effects of water and fertilizer addition on poplar-planting soil

**DOI:** 10.1128/msystems.00501-25

**Published:** 2025-06-18

**Authors:** Yimin You, Shitong Li, Xiao Li, Liran Wang, Hongxing Wang, Luping Jiang, Yanhui Peng, Zhongyi Pang, Xiyang Zhao

**Affiliations:** 1 Jilin Provincial Key Laboratory of Tree and Grass Genetics and Breeding, College of Forestry and Grassland Science, Jilin Agricultural University 85112, Changchun, China; 2 State-owned Xinmin Mechanical Forest Farm, Xinmin, China; E O Lawrence Berkeley National Laboratory, Berkeley, California, USA

**Keywords:** water and fertilizer addition, microbiological population, function, metabolism, nutrient cycle

## Abstract

While irrigation and fertilization are basic cultivation practices in poplar plantations on a global scale, the impact of these practices on the environment is not well understood. Here, we demonstrate that water-urea addition and water-compound fertilizer addition differentially impact soil ecosystems. We report that water-fertilizer addition did not significantly alter taxonomic diversity indices, but it did drive significant shifts in microbial community composition, reflected by changes in the relative abundance of specific taxa and their functional profiles. Water-urea addition reduced Proteobacteria and Actinobacteria in non-rhizosphere soils while increasing Acidobacteria and Chloroflexi. In contrast, water-compound fertilizer additions increased Proteobacteria and Actinobacteria dominance in rhizosphere soils. Water-fertilizer addition changed microbial composition and functional gene abundance linked to nitrogen and sulfur cycling. Water-urea treatment enriched denitrification genes and dissimilatory nitrate reduction genes (*napABC*) in rhizosphere soil, while water-compound fertilizer treatment enhanced nitrification (*amoABC*, *HAO*) and denitrification gene abundance in both soils. For sulfur (S) cycling, water-urea treatment favored thiosulfate oxidation genes, whereas water-compound fertilizer treatment increased assimilatory sulfate reduction genes. Multi-omics integration linked these microbial dynamics to metabolic reshaping—water-urea increased lipid and secondary metabolites in rhizosphere soils, while water-compound fertilizer additions elevated amino acid-associated metabolites in non-rhizosphere soils. Crucially, water-compound fertilizer addition reduced the gene abundance for the conversion of N_2_O to N_2_ (*nosZ*) and altered sulfur partitioning, whereas water-urea addition elevated soil NO_3_^−^ and NH_4_^+^ but depleted available K. These results highlight trade-offs between nutrient management and microbial-mediated environmental potential, guiding optimized irrigation-fertilizer strategies for sustainable poplar silviculture.

**IMPORTANCE:**

Combined irrigation and fertilizer application affect microbial community composition, and soil nitrogen and sulfur cycles (by regulating microbial composition and the abundance of genes related to nitrogen and sulfur cycles). Water-urea reduced Proteobacteria, increased Acidobacteria, and enriched denitrification genes, elevating soil NO_3_^−^/NH_4_^+^. Water-compound fertilizer boosted Proteobacteria and nitrification genes. Water-urea increased rhizosphere lipids/secondary metabolites; compound fertilizer elevated non-rhizosphere amino acids. These trade-offs between nutrient gains and environmental risks guide optimized poplar plantation management.

## INTRODUCTION

In the first half of the 20th century, global timber shortages and worldwide demand for wood products resulted in increased cultivation of fast-growing poplar varieties around the world (1). As fossil fuel resources become increasingly depleted, poplars have become increasingly important as a short-rotation woody crop for the production of bioenergy feedstocks (2). Since the late 20th century, the focus on poplar plantation has extended to ecosystem and environmental services such as phytoremediation, shelter forest construction, carbon (C) sequestration, degraded land restoration, and soil stabilization (3).

Because poplar is a fast-growing species, sustained growth requires considerable water and nutrients. Consequently, poplar is vulnerable to shortages in both (4). To maintain and improve the productivity and quality of poplar plantations, irrigation and fertilization are common practices (1, 5). However, the impact of these practices on the environment is poorly understood because most research on soil ecology in poplar plantations has focused on the influence of water or fertilizer (1, 5) rather than the cumulative impact of the two.

Abiotic and biotic factors affect the diversity and structure of soil microorganism communities. Combined irrigation and fertilizer application also affects the soil and non-target organisms within it such as bacteria and fungi. Soil-dwelling microorganisms drive nutrient biochemical cycles (e.g., carbon, nitrogen, and sulfur) and maintain soil function (6). Functional redundancy—where multiple taxa perform similar metabolic roles—serves as a critical buffer to preserve these biogeochemical functions, even amid compositional shifts in microbial communities (7). This redundancy explains why ecosystem processes like N mineralization often persist even when population dynamics fluctuate under environmental pressures (8). However, extreme perturbations (e.g., prolonged fertilization) may exceed redundancy thresholds, thereby decoupling community structure from function (9). While microbial diversity, community structure, and keystone phylotypes maintain soil function, microbial communities can be used to assess soil health and to predict function (10), metabolomic evidence increasingly reveals that functional redundancy is pathway-specific, with certain processes (e.g., denitrification) exhibiting higher redundancy than others (e.g., specialized secondary metabolism) (11). Metabolites from microbes also affect soil and rhizosphere biochemistry and can be used to characterize changes in soil microbial communities (12). Metabolites (at the molecular level) are also more sensitive to environmental stresses than traditional indicators such as microbial biomass C (13). Therefore, metabolomics provides molecular-level information on nutrient cycling and its relationship with soil microbial communities, particularly in identifying when functional redundancy fails to compensate for community disruption (14), and can be used to identify differentially enriched metabolic pathways to improve understanding of interactions between microbial communities and their environment (15).

The metagenome can be used to characterize the composition and functional diversity of the soil microbiome (15–17). Additionally, the soil metabolome contains information on many analytes such as amino acids, carbohydrates, lipids, and organic heterocyclic compounds that can be used to identify changes in the soil molecular pool (14, 18). Changes in the soil microbiome and metabolome reflect biochemical responses to irrigation and fertilizer application. The effects and mechanism of irrigation and fertilization on soil ecology within a poplar plantation have not been previously reported. We use metagenome and metabolomic profiling to identify microorganisms, enzymes, and metabolic processes occurring in the rhizosphere and adjacent soils in a poplar plantation in different irrigation and fertilizer application scenarios. The hypotheses of this study are (i) water-urea and water-compound fertilizer addition differentially influence microbial-mediated nitrogen and sulfur cycling processes in poplar plantations and (ii) the alterations in microbial community composition and functional profiles under water-fertilizer treatments are associated with changes in soil metabolite profiles and environmental risk factors.

## MATERIALS AND METHODS

### Experimental design

Research was performed at a site in the state-owned Xinmin Machinery Forest Farm, Xinmin City, Liaoning Province (122°33′53″E, 41°51′48″N). The area experiences a northern temperate monsoon climate and has an average annual temperature of 7.6°C, average annual precipitation of 608 mm, average annual sunshine of 2753.2 h, and a frost-free period of 151 d. The experimental site was established on sandy soil with a pH ranging 7.5–8.0.

The experimental forest was 10 years old and planted with Xinlin No.1 poplar at a mature-tree density of 4 m × 6 m. The water-fertilizer addition treatments were initiated in April 2023 and maintained for one full growing season (May–August 2023). The experiment involved three treatments: (−20 kPa irrigation treatment, −20 kPa irrigation +1,000 g urea tree^−1^ [water-urea treatment], and −20 kPa irrigation +1,000 g compound fertilizer tree^−1^ [water-compound fertilizer treatment]) and a control (untreated). The urea and compound fertilizer concentrations (1,000 g tree^−1^) were selected based on management guidelines of a local poplar plantation. The N fertilizer was urea (46% N), and the compound fertilizer had a N:P:K ratio of 15:15:15. To ensure comparability between treatments, the total nitrogen input was standardized: urea (46% N) provided 460 g N tree^−1^ annually, while compound fertilizer (15:15:15 NPK) delivered 150 g N, 150 g P₂O₅, and 150 g K₂O tree^−1^ annually. This design isolates irrigation effects while contrasting urea-driven N enrichment vs compound-driven NPK synergies. For each treatment and the control, there were three replicate plots (*n* = 12), with each plot containing 30 trees. Fertilizer was applied three times (early May, June, and July 2023) by ring furrow. The sample names under different treatments are shown in Table 1.

**TABLE 1:** Sample names under different treatments

	Control	Irrigation treatment	Water-urea treatment	Water-compound fertilizer treatment
Non-rhizosphere soil	CKS	HS	NS	FS
Rhizosphere soil	CKRS	HRS	NRS	SRS

### Soil samples

All sampling points were constrained within the sandy soil matrix to ensure edaphic homogeneity. No cross-soil type comparisons were conducted in this phase of the research. Non-rhizosphere soil was collected from nine randomly selected locations (≥50 cm from tree trunks) in each plot. Soil samples (0–20 cm) were collected by auger (35 mm diameter). Rhizosphere soil sampling followed a stratified protocol: nine healthy poplar trees were randomly selected, and their lateral roots (diameter 2–5 mm, length 10–15 cm) were excavated using sterilized shovels. After gently shaking the roots three times manually to remove loosely bound soil, root-adherent soil was carefully brushed off with sterile nylon brushes (bristle hardness 0.3 mm) into sterile containers. No chemical or aqueous treatments were applied to the roots during this process to preserve native microbial communities. At the same time, the poplar roots of these nine trees were collected. All samples were obtained on 10 August 2023. Soil samples were frozen in liquid nitrogen and then refrigerated at −80°C for subsequent omics, enzyme activity, and nutrient content analysis.

### Nutrient determination

Soil samples were air-dried and sieved (100 mesh). NH_4_^+^ and NO_3_^−^ contents were determined by CleverChem ONE spectrophotometer (DeChem Tech. GmbH, Hamburg, Germany) using a potassium chloride extraction method (19). Organic C was determined by an elemental analyzer (ThermoFisher, Germany) after acid treatment. Available phosphorus (P) was analyzed by alkali fusion (ThermoFisher, Germany) and available potassium (K) by flame photometer (Aoxi, China) (20, 21). Urease, leucine aminopeptidase, peroxidase, cellobiase, and acid phosphatase activities were analyzed according to manufacturer instructions (Jiancheng Bioengineering Institute, Nanjing, China). Detailed experimental steps are described in supplementary material 2.1.

### Metagenomic sequencing

#### DNA extraction, library construction, and metagenomic sequencing

DNA extract was fragmented to an average size of about 400 bp using Covaris M220 (Gene Company Limited, China) for paired-end library construction. Paired-end library was constructed using NEXTFLEX Rapid DNA-Seq (Bioo Scientific, Austin, TX, USA). Adapters containing the full complement of sequencing primer hybridization sites were ligated to the blunt end of fragments. Paired-end sequencing was performed on Illumina NovaSeq (Illumina Inc., San Diego, CA, USA) at Majorbio Bio-Pharm Technology Co., Ltd. (Shanghai, China) using NovaSeq Reagent Kits according to the manufacturer’s instructions (Illumina).

#### Sequence quality control and genome assembly

The data were analyzed on the free online platform of Majorbio Cloud Platform. Briefly, the paired-end Illumina reads were trimmed of adaptors, and low-quality reads (length <50 bp or with a quality value <20 or having N bases) were removed by fastp (22) (https://github.com/OpenGene/fastp, version 0.20.0).

Metagenomics data were assembled using MEGAHIT (23) (https://github.com/voutcn/megahit, version 1.1.2), which makes use of succinct de Bruijn graphs. Contigs with with a length ≥300 bp were selected as the final assembling result, and then the contigs were used for further gene prediction and annotation.

#### Gene prediction, taxonomy, and functional annotation

Open reading frames (ORFs) from each assembled contig were predicted using 24Prodigalv2.6.3 (https://github.com/hyattpd/Prodigal). The predicted ORFs with a length ≥100 bp were retrieved and translated into amino acid sequences using the NCBI translation table (http://www.ncbi.nlm.nih.gov/Taxonomy/taxonomyhome.html/index.cgi?chapter=tgencodes#SG1).

A non-redundant gene catalog was constructed using CD-HIT (25) (http://www.bioinformatics.org/cd-hit/, version 4.6.1) with 90% sequence identity and 90% coverage. High-quality reads were aligned to the non-redundant gene catalogs to calculate gene abundance with 95% identity using SOAPaligner (26) (https://github.com/ShujiaHuang/SOAPaligner, version 2.21).

Representative sequences of non-redundant gene catalog were aligned to NR database with an e-value cutoff of 1e^−5^ using Diamond (27) (https://github.com/bbuchfink/diamond, version 2.2.13) for taxonomic annotations. Cluster of orthologous groups of proteins (COG) annotation for the representative sequences was performed using Diamond (27) (https://github.com/bbuchfink/diamond, version 0.8.35) against the eggNOG database with an *e*-value cutoff of 1e^−5^. The KEGG annotation was conducted using Diamond (https://github.com/bbuchfink/diamond, version 0.8.35) against the Kyoto Encyclopedia of Genes and Genomes database (https://www.genome.jp/kegg/) with an *e*-value cutoff of 1e^−5^.

### LC-MS/MS analysis (non-targeted metabolomics)

#### Metabolite extraction

Fifty milligrams of solid sample was added to a 2 mL centrifuge tube, and a 6 mm diameter grinding bead was added. Four hundred microliters of extraction solution (methanol: water = 4:1 (vol:vol)) containing 0.02 mg/mL of internal standard (L-2-chlorophenylalanine) was used for metabolite extraction. Samples were ground by the Wonbio-96c (Shanghai Wanbo Biotechnology Co., LTD) frozen tissue grinder for 6 min (−10°C, 50 Hz), followed by low-temperature ultrasonic extraction for 30 min (5°C, 40 kHz). The samples were left at −20°C for 30 min, centrifuged for 15 min (4°C, 13,000 *g*), and the supernatant was transferred to the injection vial for LC-MS/MS analysis.

#### Quality control sample

As a part of the system conditioning and quality control process, a pooled quality control sample (QC) was prepared by mixing equal volumes of all samples. The QC samples were disposed and tested in the same manner as the analytic samples. It helped to represent the whole sample set, which would be injected at regular intervals (every four samples) in order to monitor the stability of the analysis.

#### LC-MS/MS analysis

The LC-MS/MS analysis of the sample was conducted on a Thermo UHPLC-Q Exactive system equipped with an ACQUITY UPLC HSS T3 (100 mm × 2.1 mm i.d., 1.8 µm; Waters, Milford, USA) at Majorbio Bio-Pharm Technology Co. Ltd. (Shanghai, China). The mobile phases consisted of 0.1% formic acid in water: acetonitrile (2:98, vol/vol) (solvent A) and 0.1% formic acid in acetonitrile (solvent B). The flow rate was 0.40 mL/min, and the column temperature was 40℃. The injection volume was 3 µL.

MS conditions were shown below. The UPLC system was coupled to a Thermo UHPLC-Q Exactive Mass Spectrometer equipped with an electrospray ionization (ESI) source operating in positive mode and negative mode. The optimal conditions were set as follows: source temperature at 425℃; sheath gas flow rate at 50 arb; Aux gas flow rate at 13 arb; ion-spray voltage floating (ISVF) at −3,500 V in negative mode and 3,500 V in positive mode, respectively; Normalized collision energy, 20-40–60 V rolling for MS/MS. Full MS resolution was 60,000, and MS/MS resolution was 7,500. Data acquisition was performed with the Data Dependent Acquisition (DDA) mode. The detection was carried out over a mass range of 70–1,050 *m*/*z*.

#### Data analysis of metabolome

The pretreatment of LC/MS raw data was performed by Progenesis QI (Waters Corporation, Milford, USA) software, and a three-dimensional data matrix in CSV format was exported. The information in this three-dimensional matrix included sample information, metabolite name, and mass spectral response intensity. Internal standard peaks, as well as any known false positive peaks (including noise, column bleed, and derivatized reagent peaks), were removed from the data matrix, deredundant, and peak pooled. At the same time, the metabolites were identified by searching database, and the main databases were the HMDB (http://www.hmdb.ca/), Metlin (https://metlin.scripps.edu/), and Majorbio Database.

The data matrix obtained by searching database was uploaded to the Majorbio cloud platform for data analysis. First, the data matrix was pre-processed, as follows: at least 80% of the metabolic features detected in any set of samples were retained. After filtering, for specific samples with metabolite levels below the lower limit of quantification, the minimum metabolite value was estimated, and each metabolic signature was normalized to the sum. To reduce the errors caused by sample preparation and instrument instability, the response intensities of the sample mass spectrometry peaks were normalized using the sum normalization method to obtain the normalized data matrix. Meanwhile, the variables of QC samples with relative standard deviation (RSD) >30% were excluded and log10 logarithmicized, to obtain the final data matrix for subsequent analysis.

Then, the R package “ropls” (Version 1.6.2) was used to perform principal component analysis (PCA) and orthogonal least partial squares discriminant analysis (OPLS-DA), and 7-cycle interactive validation evaluating the stability of the model. The metabolites with VIP >1, *P* < 0.05, were determined as significantly different metabolites based on the variable importance in the projeciton (VIP) obtained by the OPLS-DA model and the *P*-value generated by student’s *t* test.

Differential metabolites among two groups were mapped into their biochemical pathways through metabolic enrichment and pathway analysis based on KEGG database (http://www. genome.jp/kegg/). These metabolites could be classified according to the pathways they involved or the functions they performed. Enrichment analysis was used to analyze a group of metabolites in a function node whether appears or not. The principle was that the annotation analysis of a single metabolite develops into an annotation analysis of a group of metabolites. Python packages “scipy.stats” (https://docs.scipy.org/doc/scipy/) were used to perform enrichment analysis to obtain the most relevant biological pathways for experimental treatments.

Metagenomic and metabolomic analysis of soils was performed using the free online platform of the Majorbio cloud platform.

### Statistical analysis

Data are presented as mean ± standard error (SE) of biological replicates per treatment. One-way analysis of variance (one-way ANOVA) and Duncan’s multivariate range tests at *P* = 0.05 were performed on treatment data using SPSS Statistics 26 (IBM Corp., NY, USA). Chao 1, Shannon, Pielou’s, and Simpson indexes were calculated using mothur software (http://www.mothur.org/wiki/Calculators). A Wilcoxon rank sum test was used to analyze differences in α diversity between treatments (28). PCoA analysis (principal coordinate analysis) based on the Bray–Curtis distance algorithm was used to test for similarity in microbial community structure and function between samples. To identify microbial genera and metabolites that could predict treatment effects, we integrated metagenomic and metabolomic data sets through a multi-step analytical workflow. Microbial genera were prioritized based on relative abundance (in any treatment), with significant differences identified at *P* < 0.05. Metabolites were selected by ranking all differentially abundant features (ANOVA, *P* < 0.05) by their log2 fold-change magnitude (|log2FC| > 2), then retaining the top 50 of the most significantly altered metabolites. Spearman correlation analysis was exclusively applied to assess associations between the selected genera (*n* = 50) and metabolites (*n* = 50), as this non-parametric method robustly handles non-normally distributed omics data.

## RESULTS

### Effects of irrigation and fertilizer application on soil microbial communities

**Fig 1:**
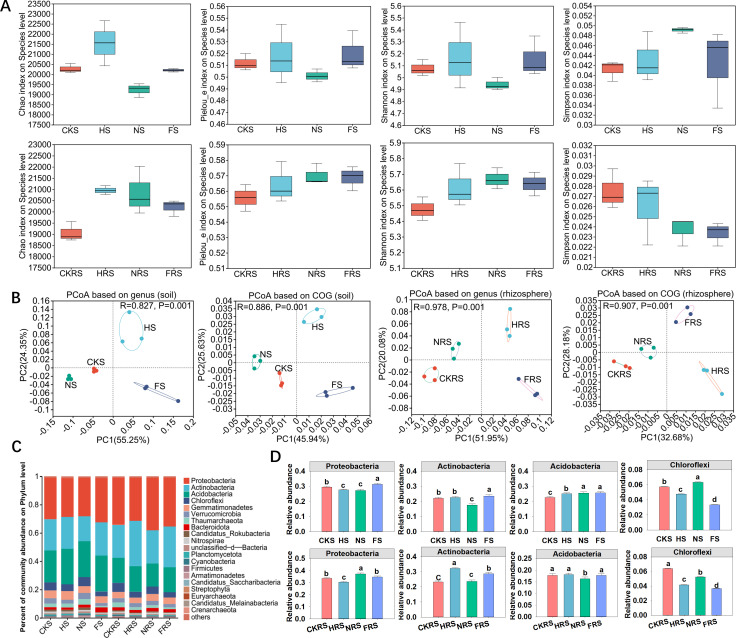
Effects of water and fertilizer addition on non-rhizosphere soil and rhizosphere soil microbiome of poplar plantation. In different treatments, (**A**) the α diversity, (**B**) PCoA principal component analysis, (**C**) microbial community composition at phylum level, and (**D**) the four phyla with the most significant changes. Bar charts with different letters indicated significant differences (*P* < 0.05). CKS, HS, NS, and FS represent the control group, irrigation treatment, water-urea treatment, and water-compound fertilizer treatment in non-rhizosphere soil, respectively. CKRS, HRS, NRS, and FRS represent the control group, irrigation treatment, water-urea treatment, and water-compound fertilizer treatment in the rhizosphere soil, respectively.

Rhizosphere soils exhibited significantly higher Shannon’s diversity and lower Simpson’s indices than non-rhizosphere soils (*P* < 0.05) (Fig. 1A; Fig. S1). PCoA revealed distinct clustering of microbial communities by treatment at both taxonomic and functional levels, driven primarily by irrigation-fertilizer interactions (*P* < 0.05) (Fig. 1B). Urea and compound fertilizer treatments induced opposing abundance shifts: urea reduced Proteobacteria/Actinobacteria in non-rhizosphere soil while elevating Acidobacteria/ Chloroflexi, whereas compound fertilizer amplified Proteobacteria/Actinobacteria (*P* < 0.05) (Fig. 1D). Key genera like *Bradyrhizobium* and *Sphingomonas* showed treatment-specific enrichment (Table S1), with urea favoring rhizosphere N-cycling taxa and compound fertilizer enhancing Streptomyces in non-rhizosphere soil.

### Potential change of soil N and S cycles

#### Soil N cycle

**Fig 2:**
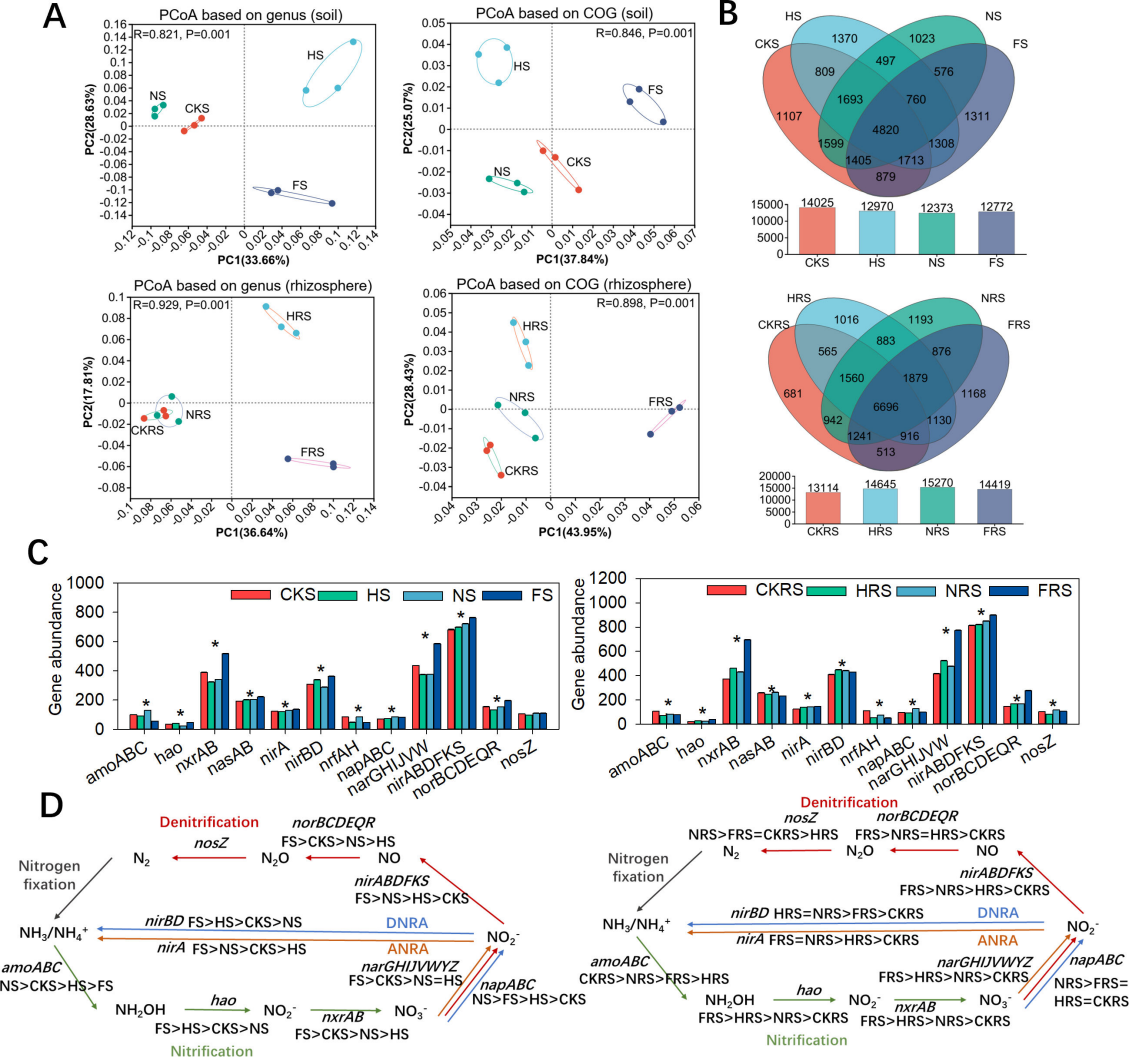
Water and fertilizer addition treatment changed the nitrogen cycle of non-rhizosphere soil and rhizosphere soil. (**A**) PCoA based on nitrogen-metabolizing genus and function (COG) levels, (**B**) Venn diagram of the number of nitrogen metabolism genes, (**C**) gene abundance in nitrogen metabolic pathways, (**D**) comparative analysis of nitrogen metabolic pathways in different treatments. CKS, HS, NS, and FS represent the control group, irrigation treatment, water-urea treatment, and water-compound fertilizer treatment in non-rhizosphere soil, respectively. CKRS, HRS, NRS, and FRS represent the control group, irrigation treatment, water-urea treatment, and water-compound fertilizer treatment in the rhizosphere soil, respectively. * represents *P* < 0.05, ** represents *P* < 0.01, and *** represents *P* < 0.001. Black, green, orange, blue, and red represent nitrogen fixation, nitrification, assimilatory nitrate reduction to ammonium (ANRA), dissimilatory nitrate reduction to ammonium (DNRA), and denitrification, respectively.

N-transformation gene profiles diverged significantly between treatments (*P* < 0.05; Fig. 2A and B; Fig. S2). Denitrification genes (*norBCDEQR*, *nirABDFKS*) dominated over nitrification genes (*amoABC*, *HAO*), reflecting oxygen-limited conditions under irrigation. Urea addition amplified dissimilatory nitrate reduction (*napABC*) and assimilatory pathways (*nirA*), increasing NH_4_^+^ in both soils (Fig. 2C and 6A). Conversely, compound fertilizer enhanced nitrification (*HAO/nxrAB*) and denitrification (*nirABDFKS/norBCDEQR*), driving NO_3_^−^ accumulation yet sustaining rhizosphere NH_4_^+^ via DNRA (Fig. 2C and D). Critical divergence emerged in terminal denitrification: urea favored *nosZ* (N_2_ production), while compound fertilizer enriched *nirABDFKS/norBCDEQR* (N_2_O precursors), suggesting fertilizer type governs N-loss pathways (Fig. 2D).

#### Soil S cycle

**Fig 3:**
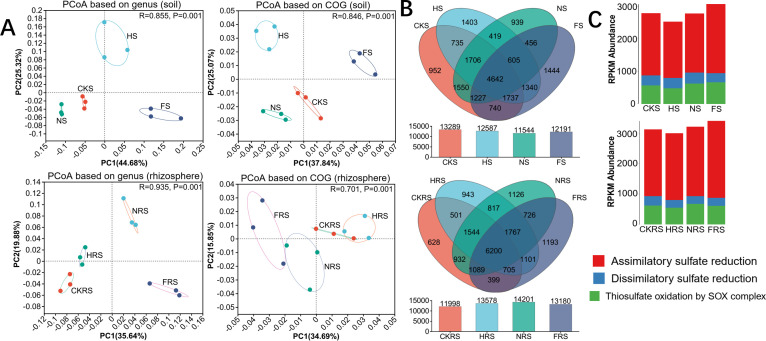
Response of sulfur metabolism to water and fertilizer addition treatment. PCoA based on genus and function in sulfur metabolism (**A**), number of genes involved in sulfur metabolism (**B**), and total gene abundance of sulfur metabolism (**C**). CKS, HS, NS, and FS represent the control group, irrigation treatment, water-urea treatment, and water-compound fertilizer treatment in non-rhizosphere soil, respectively. CKRS, HRS, NRS, and FRS represent the control group, irrigation treatment, water-urea treatment, and water-compound fertilizer treatment in the rhizosphere soil, respectively. * represents *P* < 0.05, ** represents *P* < 0.01, and *** represents *P* < 0.001.

PCoA analysis revealed changes in the genera and their functions related to S metabolism differed between soil types by treatment (*P* < 0.05) (Fig. 3A). In the water-urea and water-compound fertilizer treatments, there were significantly fewer genes involved in S metabolism in non-rhizosphere soil than there were in rhizosphere soil (Fig. 3B). The altered abundance of S-metabolism-related genes (e.g., *cysD, cysN*, *soxA, soxB*) under combined irrigation and fertilizer treatments (Fig. 3C; Fig. S3) indicates a reorganization of microbial sulfur transformation potential, which may influence sulfate availability if functional redundancy is overcome. The irrigation and urea application treatment significantly increased the total abundance of genes involved with thiosulfate oxidation by the SOX complex in soil (Fig. 3C). The irrigation and compound fertilizer application mainly increased the total abundance of genes involved with assimilatory sulfate reduction in the soil (Fig. 3C). Collectively, these results demonstrate that water-fertilizer addition does not merely amplify anaerobic S metabolism but redirects sulfur flux toward treatment-specific pathways—water-urea favoring thiosulfate oxidation by SOX complex and water-compound fertilizer enhancing assimilatory sulfate reduction (Fig. 3C).

### Effects of irrigation and fertilizer application on the soil metabolic profiles

**Fig 4:**
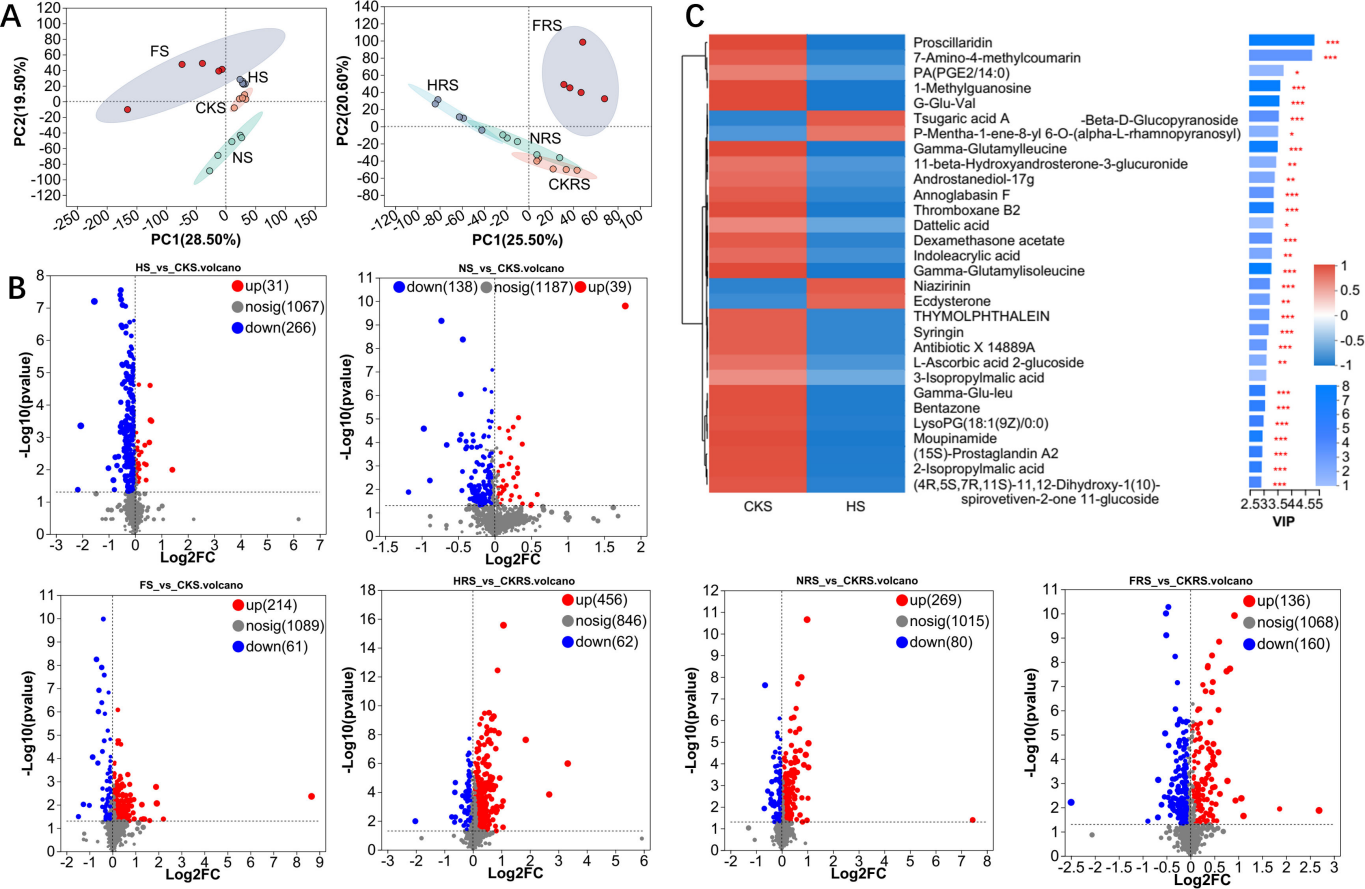
Metabolomic characteristics in different treatments. Principal component analysis of metabolites in non-rhizosphere soil and rhizosphere soil (**A**). Volcano plot of significantly different metabolites in non-rhizosphere soil and rhizosphere soil between different treatments and controls (**B**). Representative differential metabolites between control group and water treatment in non-rhizosphere soil (**C**). CKS, HS, NS, and FS represent the control group, irrigation treatment, water-urea treatment, and water-compound fertilizer treatment in non-rhizosphere soil, respectively. CKRS, HRS, NRS, and FRS represent the control group, irrigation treatment, water-urea treatment, and water-compound fertilizer treatment in the rhizosphere soil, respectively. * represents *P* < 0.05, ** represents *P* < 0.01, and *** represents *P* < 0.001.

The first and second principal components in a PCoA analysis of soil metabolic profiles separated metabolic profiles into treatments, indicating that irrigation and fertilizer application affected the low molecular weight metabolic profile of soils (Fig. 4A). In non-rhizosphere soil, compared with controls, 31 metabolites in the irrigation treatment, 39 in the water-urea treatment, and 214 in the water-compound fertilizer treatment were significantly increased, and, respectively, 266, 138, and 61 were significantly decreased (*P* < 0.05) (Fig. 4B). For rhizosphere soil (compared with controls), 456 metabolites in the irrigation treatment, 269 in the water-urea treatment, and 136 in the water-compound fertilizer treatment were significantly up-regulated, and, respectively, 62, 80, and 160 metabolites were significantly decreased (*P* < 0.05) (Fig. 4B). The effects of irrigation and water-urea treatments on soil metabolic profile appear to be more similar, and their effects on soil metabolic profile may differ from those of the water-compound fertilizer treatment. Additionally, the effects of different water and fertilizer treatments on the metabolic profiles of soil types differed significantly.

By distinguishing variable importance plot (VIP) values of the OPLS-DA model, we identified 30 representative metabolites in different treatments for which the abundance differed from values for the control group in each treatment (Fig. 4C; Fig. S4 to S8). These metabolites include lipids, nucleotides, organic heterocyclic compounds, organic acids, and other compounds that clearly separate the metabolic profiles of treatments from the control group (Fig. 4C; Fig. S4 to S8).

### Relationships between microorganisms and metabolites

The metagenome and metabolome were integrated to determine associations between differentially expressed microorganisms and metabolite changes in treatments. To determine what microorganisms and metabolites most likely explained differences between treatments, 50 representative microbial genera and 50 representative metabolites were screened. There was a significant correlation between representative microorganisms and metabolites in treated non-rhizosphere and rhizosphere soils, explaining 100% of all correlations (*P* < 0.05) (Fig. S9 to S14). However, the number of positive and negative correlations differed significantly. For example, there were 1,646 positive correlations and 854 negative correlations in non-rhizosphere soil for the irrigation treatment and 1,410 positive correlations and 1,090 negative correlations for non-rhizosphere soil in the water-urea treatment (*P* < 0.05) (Fig. S9 to S14).

Analysis revealed 50 representative gene-encoded enzymes that differed in abundance between treatments. Significant correlations were observed between these enzymes and metabolites in different treatments, accounting for 100% of all association analyses (*P* < 0.05) (Fig. S15 to S20). There was a clear correlation between changes in soil microbial communities and the metabolic profiles in different treatments.

### Comparing differences in metabolic pathways and functions

**Fig 5:**
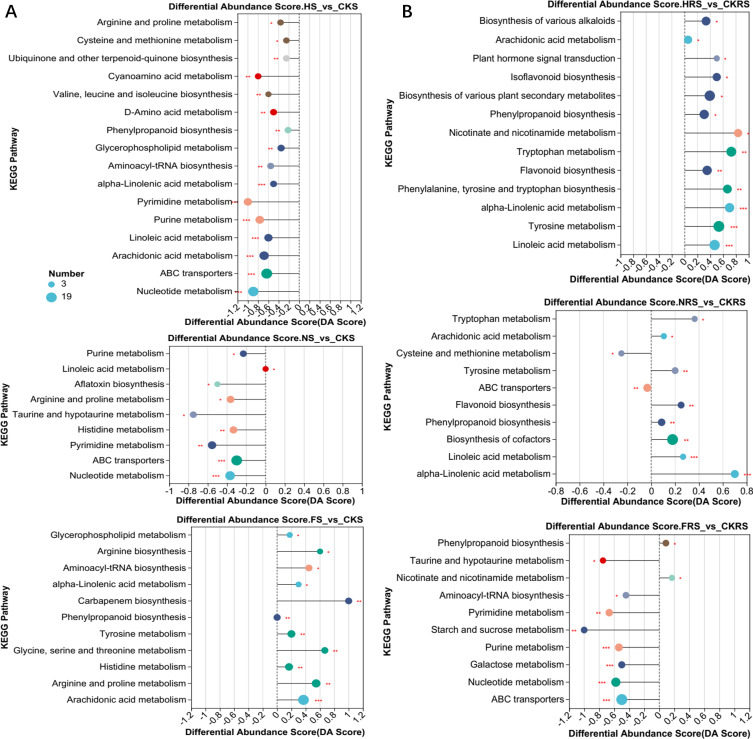
KEGG pathway enrichment was significantly different in non-rhizosphere soil (**A**) and rhizosphere soil (**B**) between different treatments and control. CKS, HS, NS, and FS represent the control group, irrigation treatment, water-urea treatment, and water-compound fertilizer treatment in non-rhizosphere soil, respectively. CKRS, HRS, NRS, and FRS represent the control group, irrigation treatment, water-urea treatment, and water-compound fertilizer treatment in the rhizosphere soil, respectively. * represents *P* < 0.05, ** represents *P* < 0.01, and *** represents *P* < 0.001.

KEGG pathway analysis revealed divergent metabolic responses between rhizosphere and non-rhizosphere soils across treatments (*P* < 0.05, Fig. 5). Irrigation significantly decreased three amino acids and four lipid metabolic pathways in non-rhizosphere soil, indicating a potential reduction in nutrient availability and lipid synthesis. Conversely, irrigation significantly increased five secondary metabolisms, three lipid, and three amino acid metabolic pathways in rhizosphere soil, suggesting enhanced metabolic activity and possibly a response to root-secreted signals (Fig. 5). The water-urea treatment significantly decreased three amino acid and two nucleotide metabolic pathways in non-rhizosphere soil, which may reflect a decreased capacity for protein synthesis and nucleic acid metabolism under this treatment. However, it significantly increased two secondary metabolisms, two lipids, and two amino acid metabolic pathways in rhizosphere soil, indicating a possible adaptation to the altered nutrient environment. The water-compound fertilizer treatment significantly increased two secondary metabolisms, three lipid, and three amino acid metabolic pathways in non-rhizosphere soil, which could be associated with enhanced nutrient uptake and metabolic processes. It significantly decreased two carbohydrate, two nucleotide, and one amino acid metabolic pathways in rhizosphere soil, possibly indicating a shift in energy metabolism and nucleic acid synthesis under this treatment (*P* < 0.05) (Fig. 5).

Affected metabolic pathways are concentrated in amino acid, lipid, and secondary metabolism. Additionally, the responses of non-rhizosphere and rhizosphere soil metabolism to irrigation treatment, water-urea addition, and water-compound fertilizer addition were opposite. These findings highlight the importance of these compounds in soil metabolism and their responsiveness to different agricultural practices. The opposite responses of non-rhizosphere and rhizosphere soil metabolism to the treatments suggest a complex interplay between soil microorganisms and plant roots.

These shifts correlated with treatment-specific enzyme alterations: urea downregulated phenylpropanoid enzymes (e.g., 4-coumarate-CoA ligase [EC:6.2.1.12]) in non-rhizosphere soil, while compound fertilizer upregulated arachidonic acid pathway components (e.g., prostaglandin synthase [EC:1.11.1.20]; Fig. S21 and S22). Such metabolic rewiring suggests root proximity modulates fertilizer-driven carbon allocation strategies.

### Relationships between water, fertilizer, and soil nutrients

**Fig 6:**
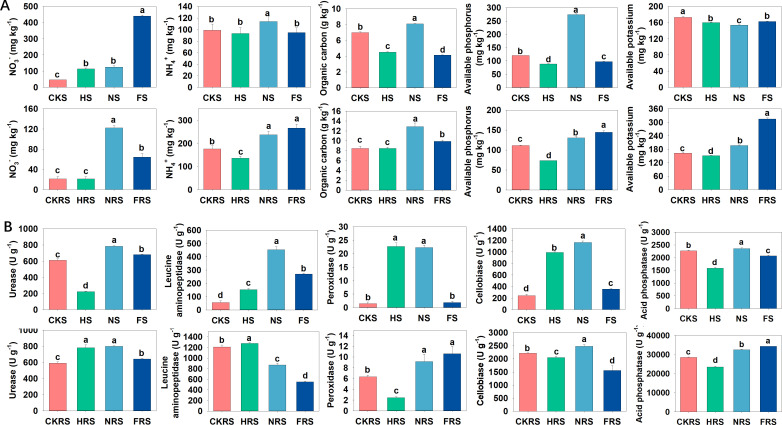
Nutrient content and enzyme activity of non-rhizosphere soil and rhizosphere soil in different treatments. NO_3_^−^, NH_4_^+^, organic carbon, available phosphorus and available potassium content (**A**). Urease, leucine aminopeptidase, peroxidase, cellobiase, and acid phosphatase activities (**B**). Bar charts with different letters indicated significant differences (*P* < 0.05). CKS, HS, NS, and FS represent the control group, irrigation treatment, water-urea treatment, and water-compound fertilizer treatment in non-rhizosphere soil, respectively. CKRS, HRS, NRS, and FRS represent the control group, irrigation treatment, water-urea treatment, and water-compound fertilizer treatment in the rhizosphere soil, respectively.

The water-urea treatment significantly increased NO_3_^−^, NH_4_^+^, organic C, and available P contents in both soil types but decreased the available K content in non-rhizosphere soil (*P* < 0.05) (Fig. 6A); significant increases in enzyme activities related to C, N, and P metabolism (including urease, peroxidase, cellobiase, and acid phosphatase) in both soil types were also apparent (*P* < 0.05) (Fig. 6B). Water-urea treatment significantly increased urease, leucine aminopeptidase, peroxidase, cellobiase, and acid phosphatase in non-rhizosphere soil. In rhizosphere soil, urease, peroxidase, cellobiase, and acid phosphatase increased significantly, while leucine aminopeptidase decreased significantly.

The water-compound fertilizer treatment significantly increased NO_3_^−^, and decreased organic C, and available P and K contents (*P* < 0.05) in non-rhizosphere soil (Fig. 6A), and significantly increased NO_3_^−^, NH_4_^+^, organic C, and available P and K contents in rhizosphere soil (*P* < 0.05) (Fig. 6B). Urease, leucine aminopeptidase, and cellobiase activities were significantly promoted in the water-compound fertilizer treatment in non-rhizosphere soil. Urease, peroxidase, and acid phosphatase activities were significantly promoted in the water-compound fertilizer treatment in rhizosphere soil, while leucine aminopeptidase (nitrogen metabolism) and cellobiase (carbon metabolism) were significantly reduced (*P* < 0.05) (Fig. 6B). Changes in soil nutrient content, enzyme activity, and metabolic genes indicate that combining irrigation and fertilizer application changes the poplar soil environment.

## DISCUSSION

### Effects of water and fertilizer on the soil microbiome

We report that the combined effects of irrigation and fertilizer application do not reduce the taxonomic evenness or diversity of soil microbial communities. Compared with non-rhizosphere soil, the significantly higher Shannon index and lower Simpson index in rhizosphere soils suggest greater microbial diversity in the rhizosphere, consistent with the enrichment of root exudates and nutrients in this microenvironment (29). Similar studies have shown that rhizosphere microbial communities are shaped by plant-derived carbon inputs and redox dynamics, which enhance niche differentiation (30). For example, irrigation practices can alter nitrogen availability and oxygen levels, indirectly driving rhizosphere microbial diversity (31). The lack of significant differences in Chao and Pielou indices between treatments may reflect the resilience of microbial a-diversity to short-term water-fertilizer interventions (32). Zhou et al. reported that a-diversity in soils was less sensitive to fertilization than community composition, which aligns with our findings (33).

An increasing number of studies have demonstrated the importance of soil microbial communities in maintaining ecosystem functions, including organic matter decomposition and nutrient cycling (9, 34). We report that irrigation and fertilizer application changed the soil’s microbial community, which was dominated by Proteobacteria, Actinobacteria, Acidobacteria, and Chloroflexi. Results of PCoA at the genus level and functional classification are consistent with the notion that water and nutrient inputs can alter the soil’s nutrient environment and microbial community structure (35). For instance, the high available N or P contents usually promote plant growth and total underground C input (36). Because soil microorganisms are usually limited by C, increasing C availability may stimulate microbial reproduction (37). Combining water with fertilizer significantly affected the soil nutrient environment, including NO_3_^−^, NH_4_^+^, organic C, and available P and K. Change in soil may benefit certain eutrophic taxa (38, 39). While previous studies have shown that certain Proteobacteria and Actinobacteria (e.g., urease-encoding taxa) thrive in urea-rich environments (40), our results revealed that the relative abundance of these phyla was lower in the water-urea treatment but higher in the water-compound fertilizer treatment. This suggests that community-level responses to nutrient inputs may not solely depend on enzymatic capacity (e.g., urease activity) but could also reflect competitive interactions, pH sensitivity, or functional tradeoffs between copiotrophic and oligotrophic strategies under contrasting fertilization regimes (41, 42). The relative abundances of Acidobacteria and Chloroflexi were greater in the water-urea treatment. This pattern may reflect their ecological resilience to urea-induced environmental changes (e.g., pH fluctuations or ammonia accumulation) or their capacity to metabolize nitrogen-enriched organic byproducts generated during urea hydrolysis, rather than a direct preference for urea itself (43, 44). Further studies targeting genomic or functional traits are needed to clarify their substrate utilization strategies under such conditions. Observed changes in microbial community composition raise questions about functional redundancy within these communities. Functional redundancy suggests that multiple taxa may perform similar functions, which could buffer the ecosystem against perturbations (45). However, the phylogenetic changes observed in our study may have implications for the resilience and functionality of the soil microbial community, which warrants further investigation.

### Effects of irrigation and fertilizer application on metabolic cycles

Irrigation-fertilizer addition induced profound redox-mediated shifts in nitrogen cycling. The upregulation of denitrification genes (*norBCDEQR*, *nirABDFKS*) over nitrification genes (*amoABC*, *HAO*) (Fig. 2C) mirrors observations in waterlogged agroecosystems, confirming oxygen limitation as a primary driver (46, 47). However, we uniquely demonstrate divergent denitrification endpoints: urea promoted *nosZ*-mediated N_2_ production, consistent with Tian et al. (48) where water-urea enhanced *nosZ* expression in soils (48), while water-compound fertilizer amplified *nirABDFKS/norBCDEQR*-driven N_2_O accumulation, mirroring Shan et al. (2021) who linked nitrate-based fertilizers to N_2_O emission hotspots (49). This bifurcation highlights a critical tradeoff: urea favors NH_4_^+^ retention but risks partial nitrification (rhizospheric NO_3_^−^), whereas compound fertilizer enhances nitrification but exacerbates greenhouse gas emissions.

Similar redox-driven specialization occurred in sulfur cycling. The water-urea treatment upregulated SOX complex genes (*soxA/B*) for thiosulfate oxidation, consistent with Friedrich et al. (50) who reported S oxidation to be mediated under transient anoxia (50). Conversely, compound fertilizer enhanced assimilatory sulfate reduction (*cysD/N*), previously observed by Zhang et al. (12) where S incorporation was prioritized under high nutrient loads (51). These results indicate that urea promotes energy-generating sulfur oxidation, while compound fertilizer redirects sulfur flux toward microbial biomass—a tradeoff that may reduce plant-available sulfate, though rhizosphere resilience likely buffers this limitation through root exudate-driven microbial recruitment (52).

Metabolic network analysis revealed treatment-compartment interplay. In rhizosphere soil, urea upregulated lipid biosynthesis genes (Fig. S6), correlating with Actinobacteria’s carbon storage strategies under nitrogen excess (53). Simultaneously, compound fertilizer upregulated phenylpropanoid biosynthesis, a response primarily linked to oxidative stress mitigation through enhanced antioxidant metabolite production (e.g., flavonoids and lignin precursors), as shown in Dixon et al. (54), who demonstrated phenylpropanoid pathway activation under nitrogen-induced redox imbalance (54). These suggested microbial metabolic flexibility in balancing growth demands and environmental pressures.

Functional microbial taxa orchestrated cross-cycle interactions. *Nocardioides* and *Streptomyces* (Actinobacteria) mediated N/S transformations and secondary metabolite synthesis, consistent with their roles in organic matter decomposition and nutrient cycling (55–58). Proteobacteria (e.g., *Nitrospira*, denitrifiers) dominated nitrification–denitrification, aligning with global surveys identifying Proteobacteria as key N-cycling drivers (59). These taxa collectively demonstrate functional redundancy—a critical feature for maintaining soil functionality under agricultural perturbations.

While this study advances understanding of irrigation-fertilizer effects on soil microbiomes, key limitations exist. Site-specific findings from poplar plantations require validation in diverse ecosystems (e.g., acidic soils, arid regions) to assess the generalizability of urea’s NH_4_^+^ retention vs compound fertilizer’s N_2_O risks. Although omics data revealed functional potential, direct flux measurements (e.g., ^15^N tracing, N_2_O emissions) are needed to confirm process rates. Future priorities include multi-year, multi-site trials evaluating microbial-metabolic trajectory stability; integrated isotope-gas flux analyses quantifying treatment-specific N/S cycling rates; mechanistic studies dissecting root exudate roles via synthetic microbial communities. Resolving these gaps will refine precision nutrient strategies for sustainable poplar cultivation.

### Conclusion

We use an integrated multi-omics framework to identify and validate changes in microbial and soil metabolomes associated with combined irrigation and fertilizer application. Combining irrigation and fertilizer application does not significantly affect soil microbial taxonomic diversity, richness, or evenness, but it does change community composition and the relative abundance of core taxa, and because it also changes soil nutrient environments, the metabolic function of microorganisms and soil nutrient cycles (e.g., amino acid, lipid, secondary, and N and S metabolism). We report contrasting trends of combining irrigation and fertilizer application on amino acid, lipid, and secondary metabolism in non-rhizosphere and rhizosphere soils. Additionally, compared with control and simple irrigation treatments, combining irrigation and compound fertilizer application promoted denitrification and S-reduction processes in both soil types, and increased greenhouse gas N_2_O emissions and soil S loss. Combined irrigation and urea application increased N_2_ emissions. Overall, these results reveal the potential mechanism by which the soil microbiome and metabolome respond to combined irrigation and fertilizer application, thereby improving our understanding of some environmental risks associated with common poplar plantation forestry practices.

## Data Availability

Metagenomic raw sequencing reads were deposited in the NCBI Sequence Read Archive (SRA) database under accession number PRJNA1123506. Metabolomic data were submitted to the MetaboLights repository under accession number MTBLS12375. The nutrient content and enzyme activity data in Fig. 6 are provided in Data S1 and S2. The data sets used and/or analyzed during the current study are available from the corresponding author on reasonable request.

## SUPPLEMENTAL MATERIAL

Supplemental data sets msystems.00501-25-s0001.xlsx Data S1 and S2.

Supplemental material msystems.00501-25-s0002.docx Additional experimental details, Fig. S1 to S22, and Table S1.

ASM does not own the copyrights to Supplemental Material that may be linked to, or accessed through, an article. The authors have granted ASM a non-exclusive, world-wide license to publish the Supplemental Material files. Please contact the corresponding author directly for reuse.
