# Supplementary material for: Ecological effects of water and fertilizer addition on poplar-planting soil
Source: mSystems. 2025 Jun 18;10(7):e00501-25. doi: 10.1128/msystems.00501-25 (PMC12282158; doi:10.1128/msystems.00501-25)
Supplement: Supplemental material — Additional experimental details, Fig. S1 to S22, and Table S1. [file msystems.00501-25-s0002.docx]

**Supplementary materials**

**2.1 Nutrient determination**

Soil samples were air-dried and screened at 0.15 mm. NH_4_^+^ and NO_3_^-^ were extracted with 2 M KCl at a soil/extractant ratio of 1:5 after shaking for 60 min at 250 rpm and 25℃ (1). Then the extract was filtered through double loop quantitative filter paper (Whatman, China) and was analyzed on a CleverChem ONE spectrophotometer (Alliance company, France) by extraction with KCl solution - automated method with segmented flow analysis (1, 2). Available phosphorus was analyzed by spectrophotometric determination after alkali fusion (ThermoFisher, Germany) (3). Rapidly available potassium content was measured by flame photometer (Aoxi company, China). Organic carbon was determined using element analyzer (ThermoFisher, Germany) (4).

Table S1 Relative abundance of microbial genera in different treatments

| Genus | CKRS | HRS | NRS | FRS | CKS | HS | NS | FS |
| --- | --- | --- | --- | --- | --- | --- | --- | --- |
| *Bradyrhizobium* | 0.041 | 0.048 | 0.059 | 0.045 | 0.024 | 0.026 | 0.029 | 0.048 |
| *Sphingomonas* | 0.034 | 0.025 | 0.042 | 0.027 | 0.020 | 0.010 | 0.016 | 0.016 |
| *Nocardioides* | 0.025 | 0.027 | 0.024 | 0.033 | 0.018 | 0.013 | 0.015 | 0.023 |
| *Streptomyces* | 0.013 | 0.025 | 0.020 | 0.016 | 0.007 | 0.018 | 0.005 | 0.006 |
| *Mycobacterium* | 0.010 | 0.013 | 0.012 | 0.010 | 0.005 | 0.005 | 0.004 | 0.007 |
| *Solirubrobacter* | 0.009 | 0.009 | 0.009 | 0.009 | 0.006 | 0.004 | 0.006 | 0.005 |
| *Nitrospira* | 0.008 | 0.005 | 0.008 | 0.009 | 0.008 | 0.004 | 0.008 | 0.006 |
| *Arthrobacter* | 0.009 | 0.007 | 0.006 | 0.010 | 0.006 | 0.002 | 0.005 | 0.005 |
| *Steroidobacter* | 0.009 | 0.004 | 0.007 | 0.004 | 0.006 | 0.002 | 0.005 | 0.003 |
| *Pseudonocardia* | 0.005 | 0.009 | 0.007 | 0.005 | 0.003 | 0.002 | 0.003 | 0.003 |
| *Pseudolabrys* | 0.003 | 0.004 | 0.005 | 0.005 | 0.005 | 0.007 | 0.006 | 0.005 |
| *Mesorhizobium* | 0.005 | 0.007 | 0.005 | 0.006 | 0.003 | 0.002 | 0.004 | 0.003 |
| *Reyranella* | 0.006 | 0.004 | 0.006 | 0.005 | 0.004 | 0.002 | 0.005 | 0.003 |
| *Paraburkholderia* | 0.002 | 0.007 | 0.006 | 0.005 | 0.001 | 0.002 | 0.001 | 0.008 |
| *Mycolicibacterium* | 0.005 | 0.004 | 0.007 | 0.004 | 0.002 | 0.001 | 0.002 | 0.003 |
| *Rhodanobacter* | 0.001 | 0.001 | 0.001 | 0.023 | 0.000 | 0.001 | 0.000 | 0.001 |
| *Niastella* | 0.002 | 0.003 | 0.003 | 0.002 | 0.001 | 0.001 | 0.001 | 0.013 |
| *Actinoplanes* | 0.004 | 0.004 | 0.004 | 0.003 | 0.003 | 0.003 | 0.003 | 0.003 |

**Supplementary Figures**

Fig. S1 α diversity of non-rhizosphere soil and rhizosphere soil in different treatments. CKS, HS, NS and FS represent the control group, irrigation treatment, water-urea treatment and water-compound fertilizer treatment in non-rhizosphere soil respectively. CKRS, HRS, NRS and FRS represent the control group, irrigation treatment, water-urea treatment and water-compound fertilizer treatment in the rhizosphere soil, respectively. The * represents *p*<0.05, ** represents *p*<0.01, and *** represents *p*<0.001.

Fig. S2 Total gene abundance of nitrogen metabolism in different treatments. CKS, HS, NS and FS represent the control group, irrigation treatment, water-urea treatment and water-compound fertilizer treatment in non-rhizosphere soil, respectively. CKRS, HRS, NRS and FRS represent the control group, irrigation treatment, water-urea treatment and water-compound fertilizer treatment in the rhizosphere soil, respectively. DNRA：Dissimilatory nitrate reduction to ammonium. ANRA: assimilatory nitrate reduction to ammonium.

Fig. S3 Response of sulfur metabolism genes in non-rhizosphere soil (A) and rhizosphere soil (B) to water and fertilizer coupling treatment. CKS, HS, NS and FS represent the control group, irrigation treatment, water-urea treatment and water-compound fertilizer treatment in non-rhizosphere soil respectively. CKRS, HRS, NRS and FRS represent the control group, irrigation treatment, water-urea treatment and water-compound fertilizer treatment in the rhizosphere soil, respectively.


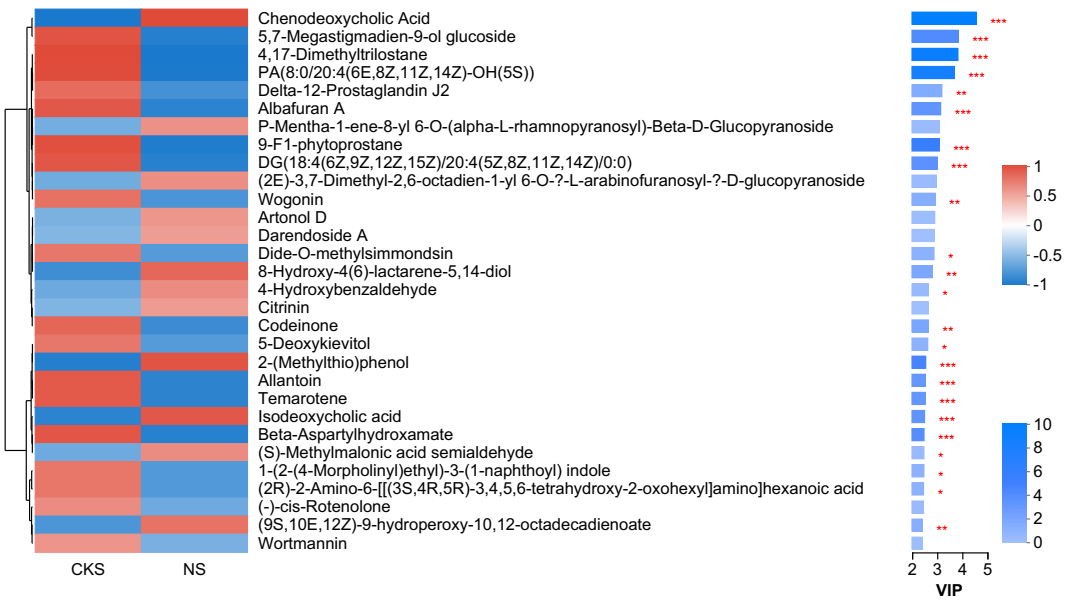


Fig. S4 Representative differential metabolites in non-rhizosphere soil under water-urea coupling (NS) treatment compared with control (CKS). The * represents *p*<0.05, ** represents *p*<0.01, and *** represents *p*<0.001.


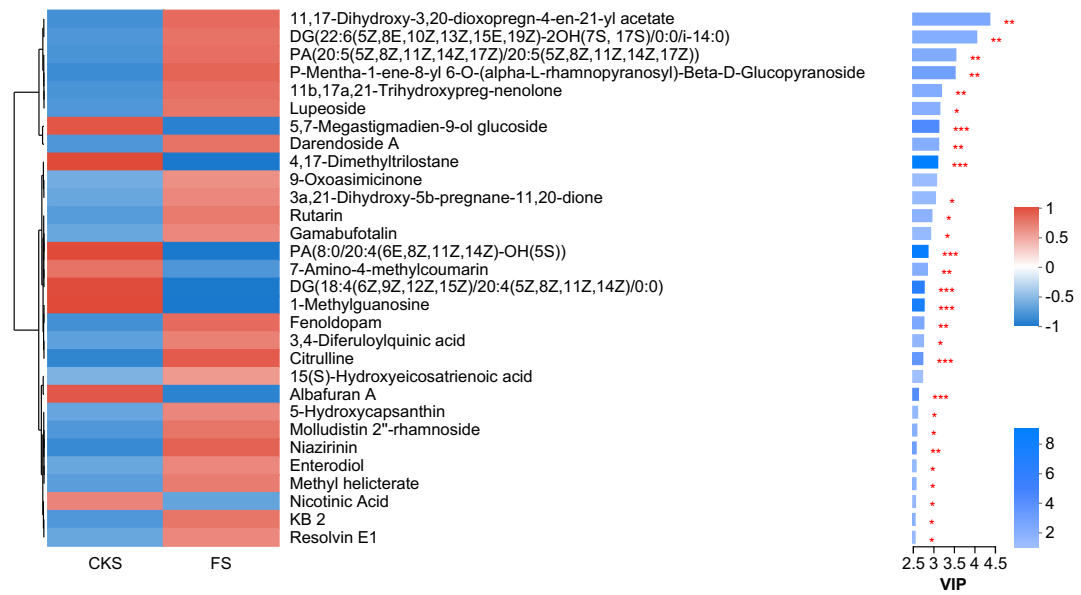


Fig. S5 Compared with the control (CKS), representative differential metabolites in non-rhizosphere soil under water-compound fertilizer coupling (FS) treatment. The * represents *p*<0.05, ** represents *p*<0.01, and *** represents *p*<0.001.


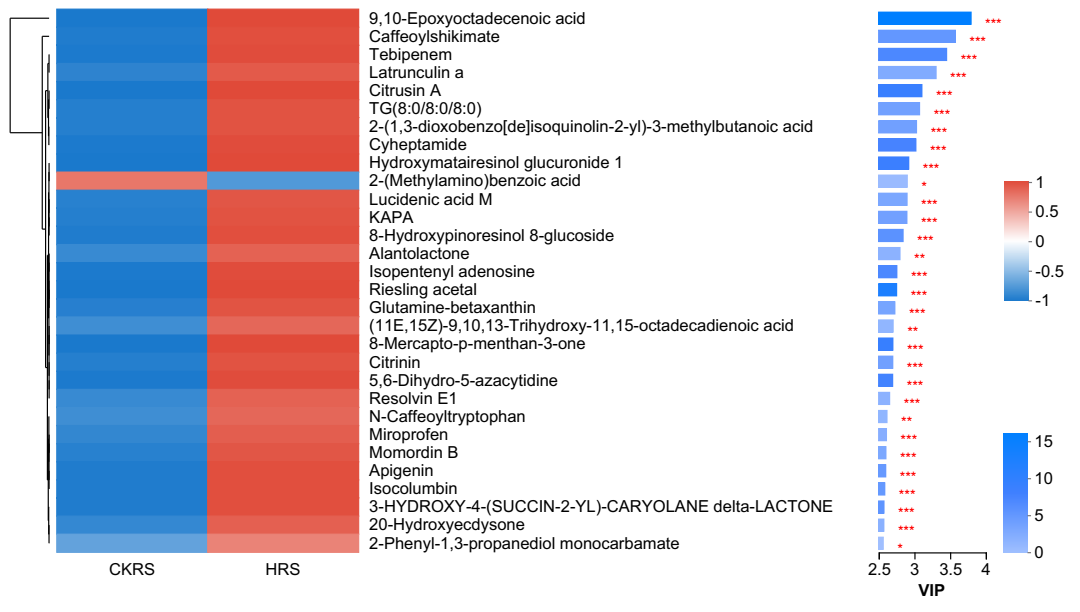


Fig. S6 Representative differential metabolites in rhizosphere soil under irrigation (HRS) treatment compared with control (CKRS). The * represents *p*<0.05, ** represents *p*<0.01, and *** represents *p*<0.001.


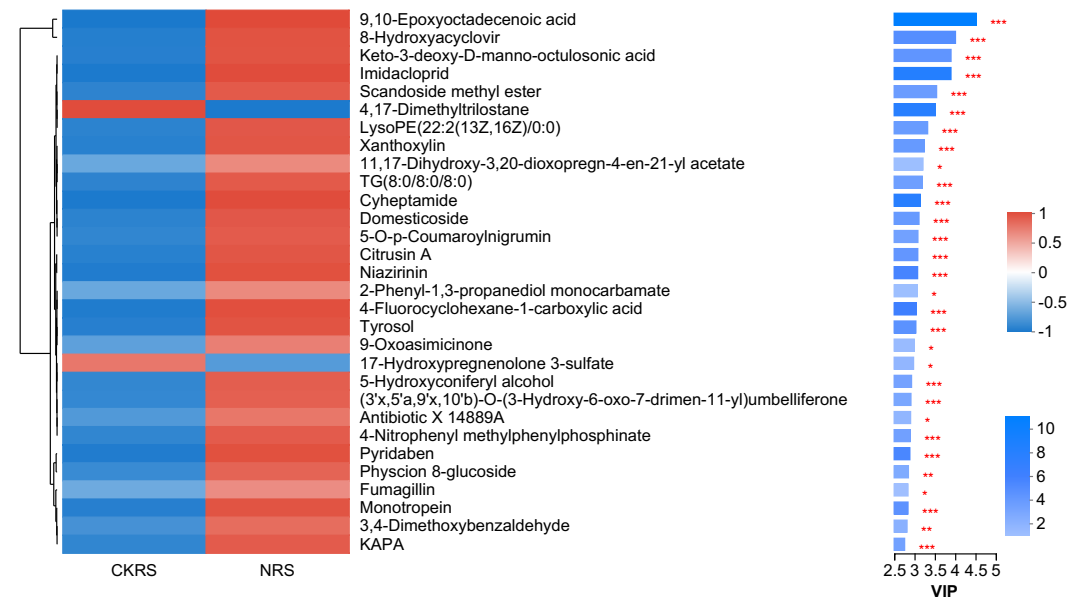


Fig. S7 Representative differential metabolites in rhizosphere soil under water-urea coupling (NRS) treatment compared with control (CKRS). The * represents *p*<0.05, ** represents *p*<0.01, and *** represents *p*<0.001.


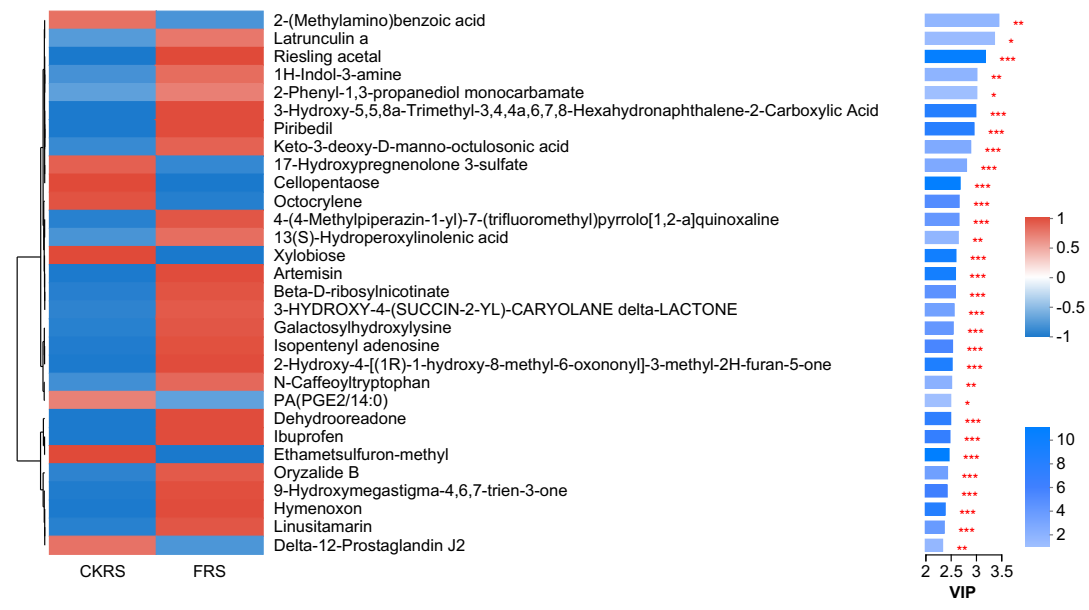


Fig. S8 Compared with the control (CKRS), representative differential metabolites in rhizosphere soil under water-compound fertilizer coupling (FRS) treatment. The * represents *p*<0.05, ** represents *p*<0.01, and *** represents *p*<0.001.


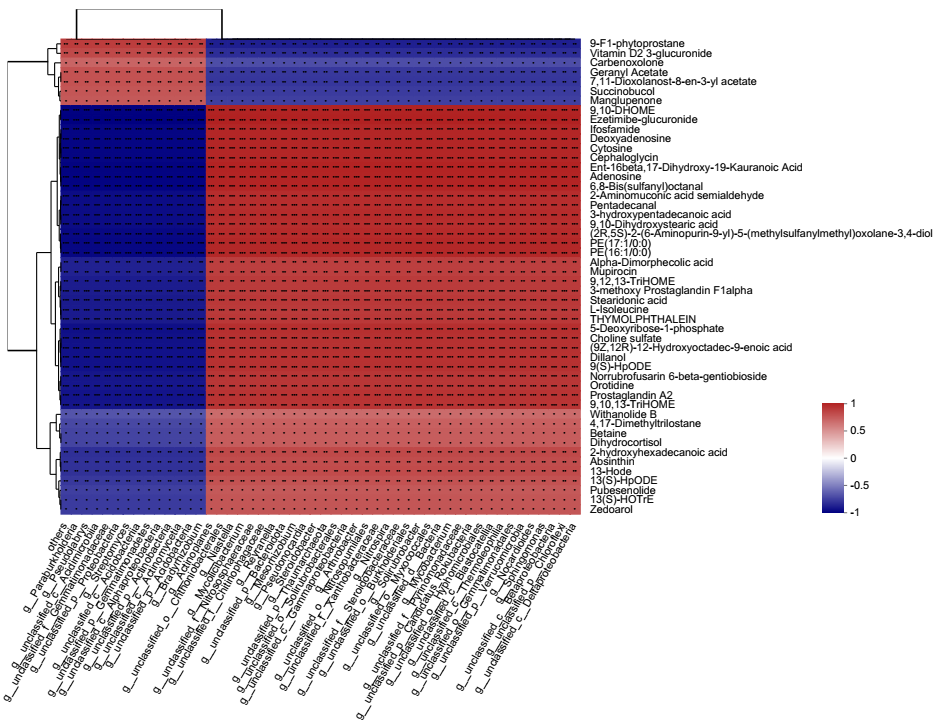


Fig. S9 Correlation between 50 representative genera and 50 significantly differential metabolites in microorganisms in non-rhizosphere soil treated with irrigation. The * represents *p*<0.05, ** represents *p*<0.01, and *** represents *p*<0.001.


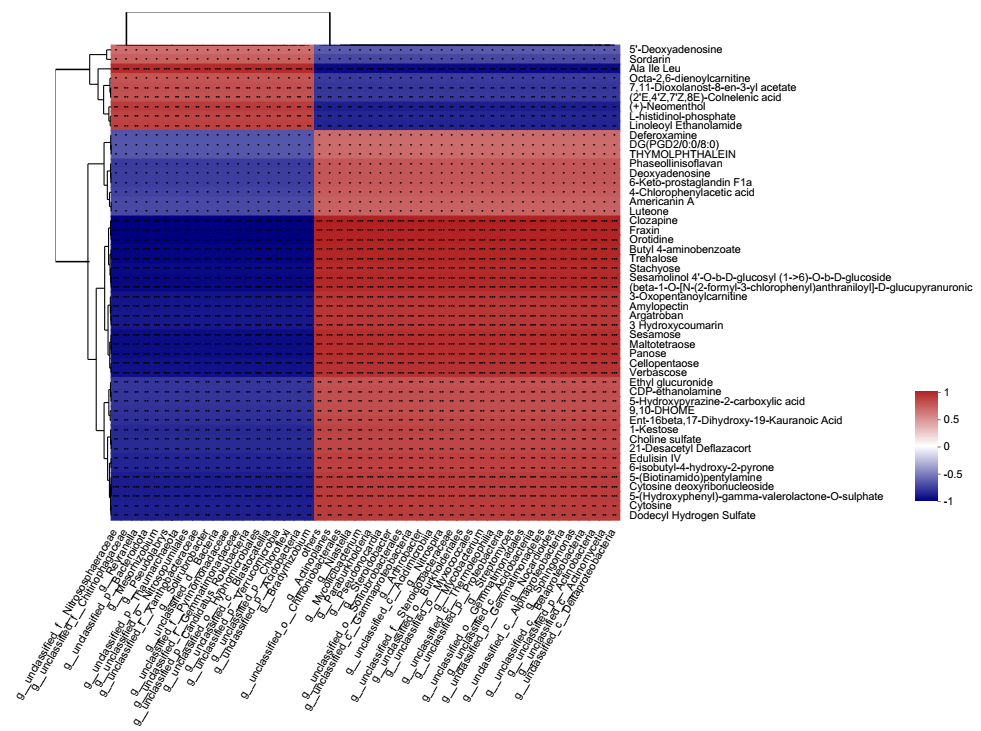


Fig. S10 Correlation between 50 representative genera and 50 significantly differential metabolites in microorganisms in non-rhizosphere soil treated with water-urea. The * represents *p*<0.05, ** represents *p*<0.01, and *** represents *p*<0.001.


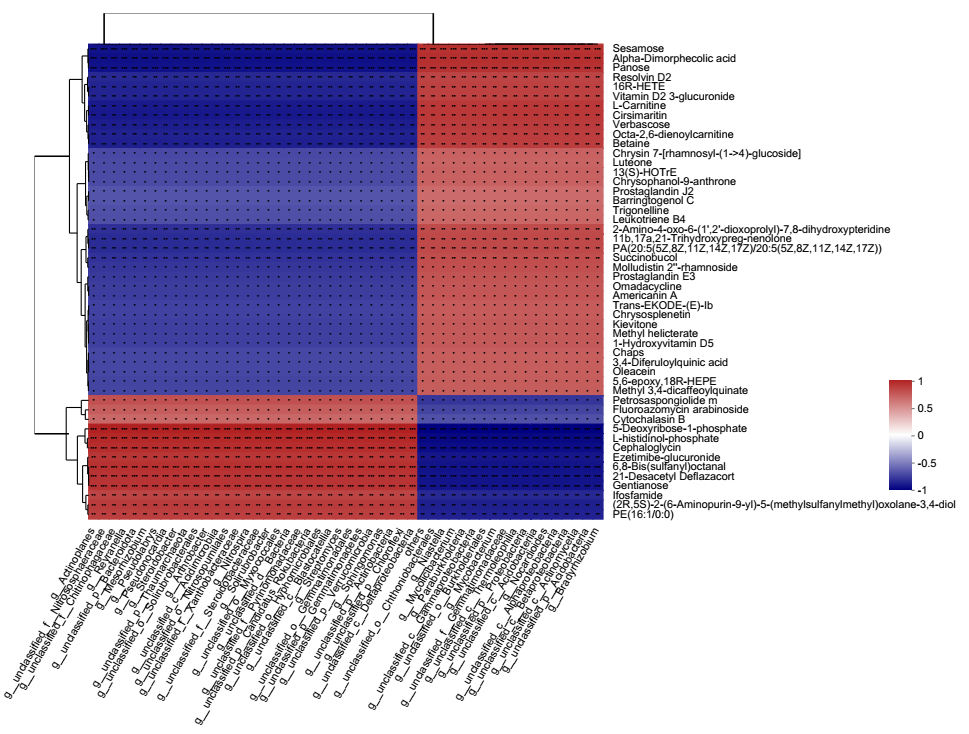


Fig. S11 Correlation between 50 representative genera and 50 significantly differential metabolites in microorganisms in non-rhizosphere soil treated with water-compound fertilizer. The * represents *p*<0.05, ** represents *p*<0.01, and *** represents *p*<0.001.


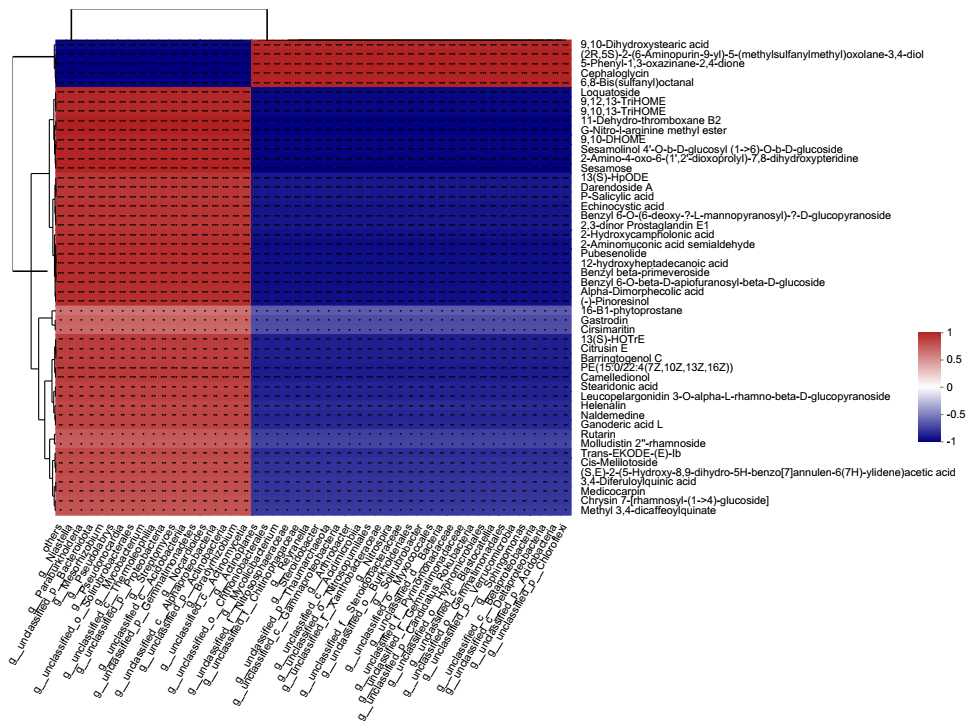


Fig. S12 The correlation between 50 representative genera and 50 significantly differential metabolites in rhizosphere soil treated with irrigation. The * represents *p*<0.05, ** represents *p*<0.01, and *** represents *p*<0.001.


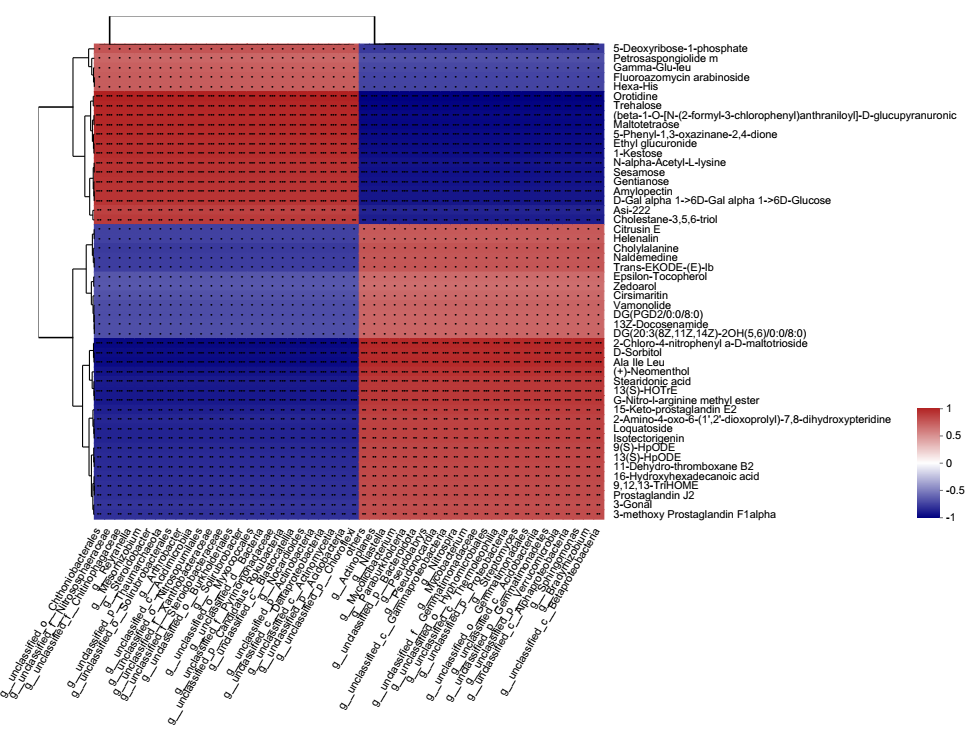


Fig. S13 Correlation between 50 representative bacterial genera and 50 significantly differential metabolites in rhizosphere soil of water-urea treatment. The * represents *p*<0.05, ** represents *p*<0.01, and *** represents *p*<0.001.


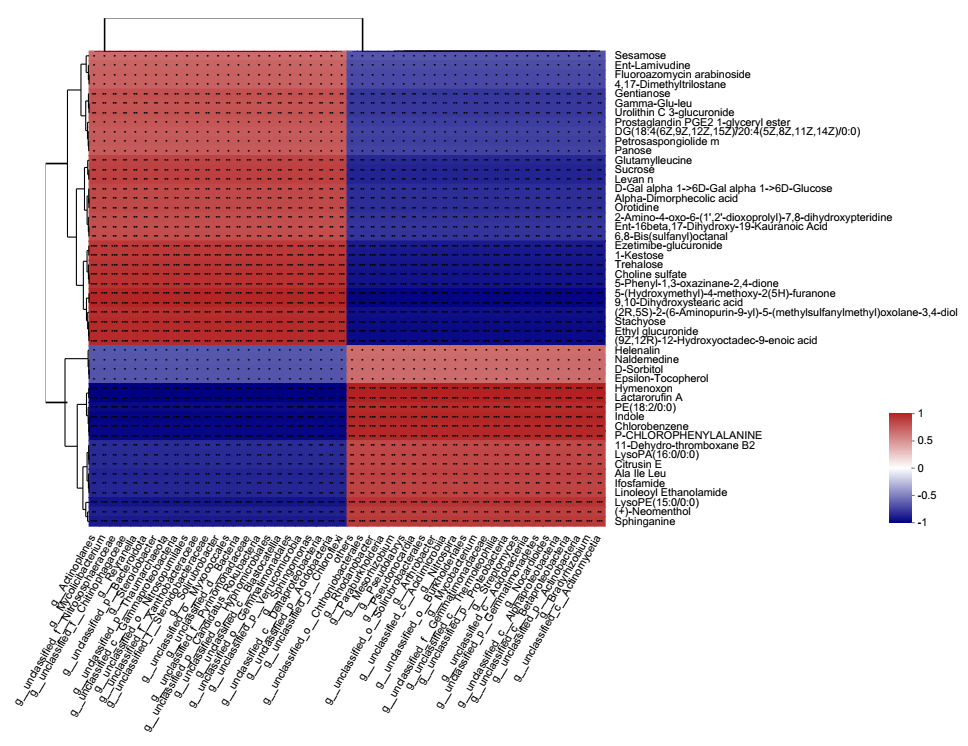


Fig. S14 Correlation between 50 representative genera and 50 significantly differential metabolites in rhizosphere soil treated with water-compound fertilizer. The * represents *p*<0.05, ** represents *p*<0.01, and *** represents *p*<0.001.


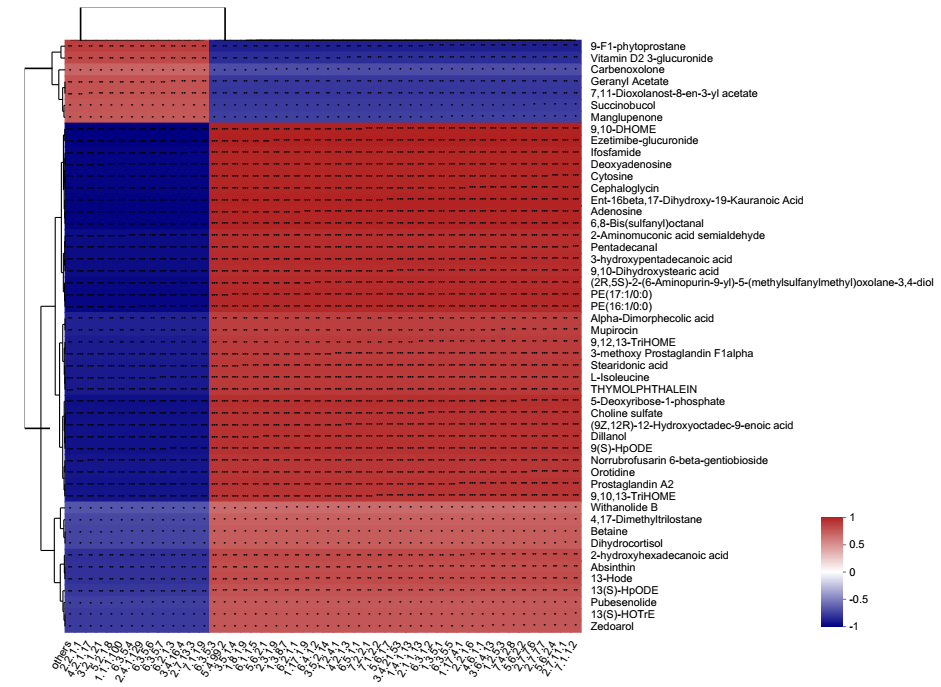


Fig. S15 The correlation between the abundance of 50 representative enzymes in microorganisms and 50 significantly differential metabolites in non-rhizosphere soil treated with irrigation. The * represents *p*<0.05, ** represents *p*<0.01, and *** represents *p*<0.001. Enzyme Commission (EC) Numbers can be inquired in the website: https://www.brenda-enzymes.org/all_enzymes.php.


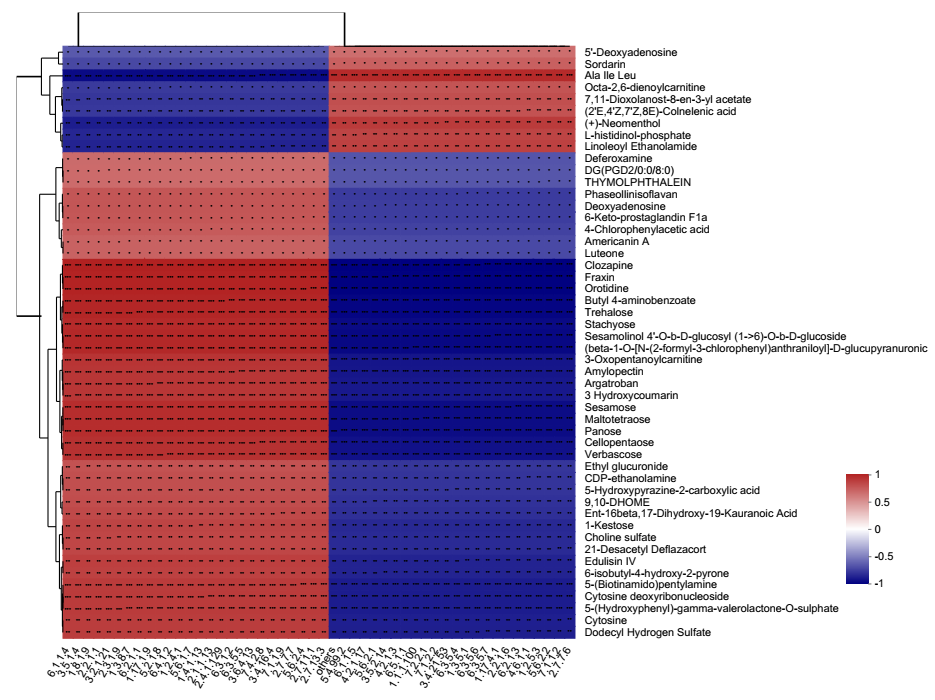


Fig. S16 Correlation between the abundance of 50 representative enzymes in microorganisms and 50 significantly differential metabolites in non-rhizosphere soil treated with water-urea. The * represents *p*<0.05, ** represents *p*<0.01, and *** represents *p*<0.001. Enzyme Commission (EC) Numbers can be inquired in the website: https://www.brenda-enzymes.org/all_enzymes.php.


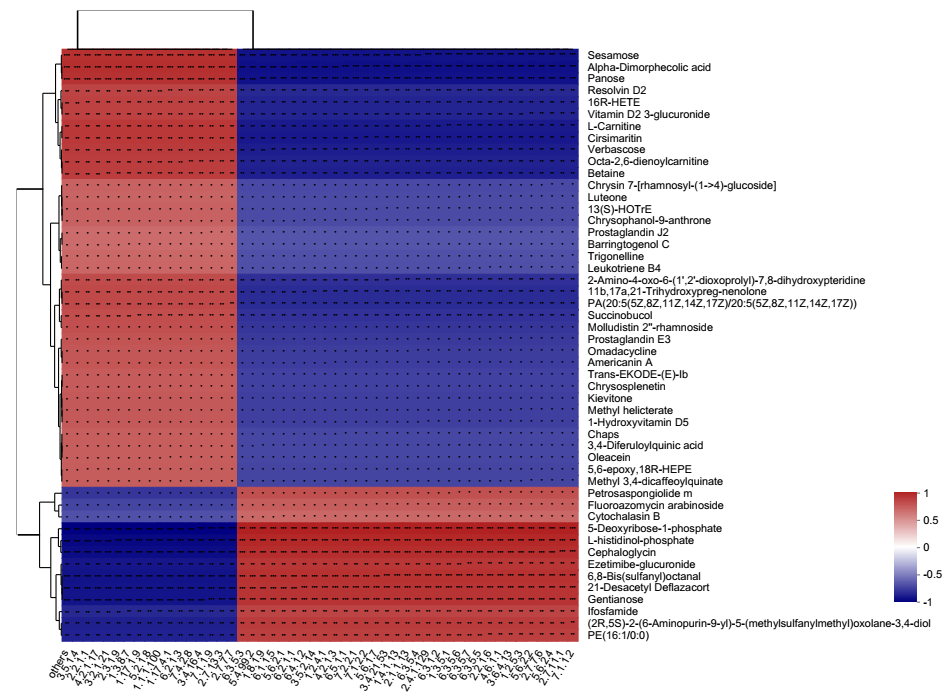


Fig. S17 Correlation between the abundance of 50 representative enzymes in microorganisms and 50 significantly differential metabolites in non-rhizosphere soil treated with water-compound fertilizer. The * represents *p*<0.05, ** represents *p*<0.01, and *** represents *p*<0.001. Enzyme Commission (EC) Numbers can be inquired in the website: https://www.brenda-enzymes.org/all_enzymes.php.


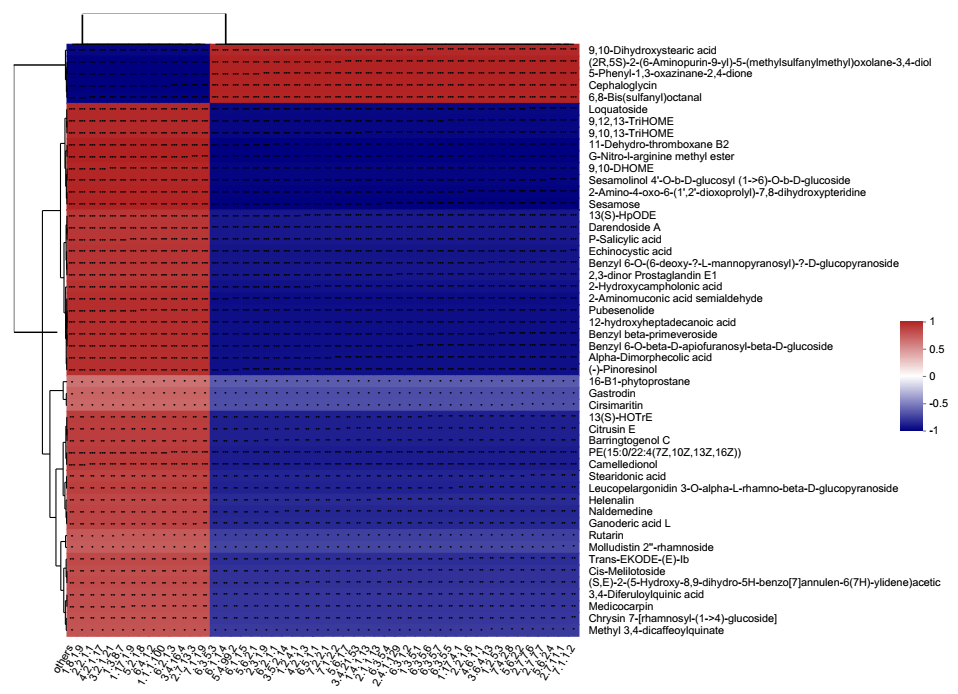


Fig. S18 The correlation between the abundance of 50 representative enzymes in microorganisms and 50 significantly differential metabolites in rhizosphere soil treated with irrigation. The * represents *p*<0.05, ** represents *p*<0.01, and *** represents *p*<0.001. Enzyme Commission (EC) Numbers can be inquired in the website: https://www.brenda-enzymes.org/all_enzymes.php.


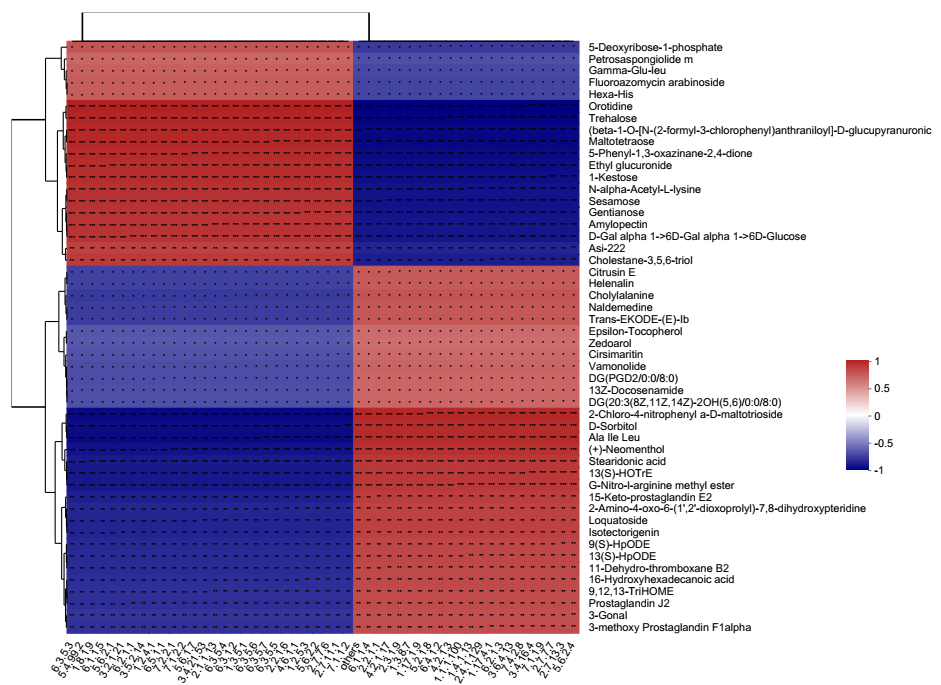


Fig. S19 Correlation between the abundance of 50 representative enzymes and 50 significantly differential metabolites in rhizosphere soil treated with water-urea. The * represents *p*<0.05, ** represents *p*<0.01, and *** represents *p*<0.001. Enzyme Commission (EC) Numbers can be inquired in the website: https://www.brenda-enzymes.org/all_enzymes.php.


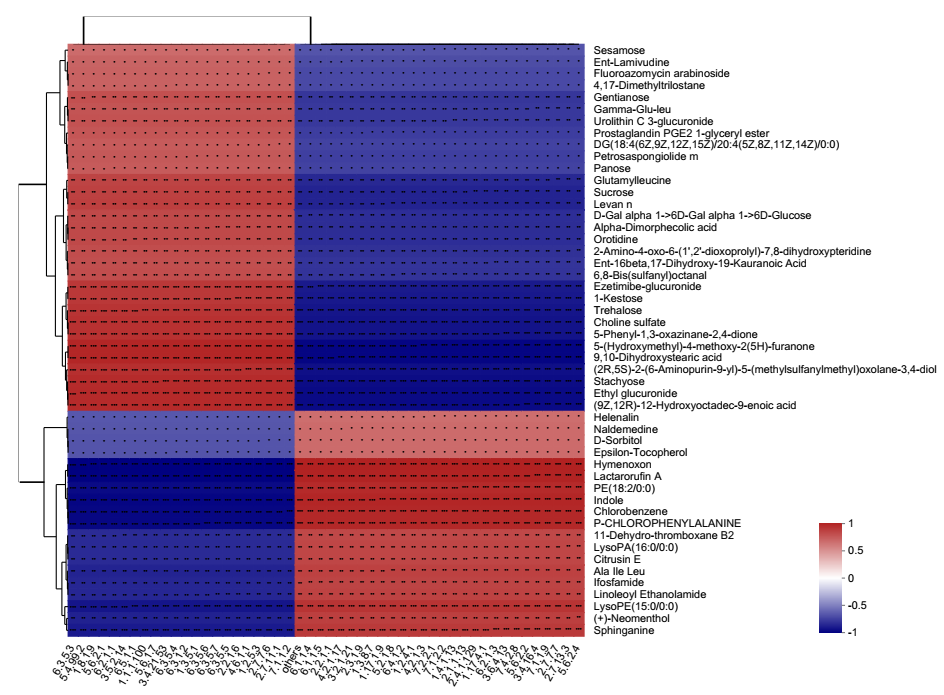


Fig. S20 Correlation between the abundance of 50 representative enzymes in microorganisms and 50 significantly differential metabolites in rhizosphere soil treated with water-compound fertilizer. The * represents *p*<0.05, ** represents *p*<0.01, and *** represents *p*<0.001. Enzyme Commission (EC) Numbers can be inquired in the website: https://www.brenda-enzymes.org/all_enzymes.php.

Fig. S21 Phenylpropanoid metabolic pathway. CKS, HS, NS and FS represent the control group, irrigation treatment, water-urea treatment and water-compound fertilizer treatment in non-rhizosphere soil respectively. CKRS, HRS, NRS and FRS represent the control group, irrigation treatment, water-urea treatment and water-compound fertilizer treatment in the rhizosphere soil, respectively. Enzyme Commission (EC) Numbers can be inquired in the website: https://www.brenda-enzymes.org/all_enzymes.php.

Fig. S22 Arachidonic acid metabolic pathway. CKS, HS, NS and FS represent the control group, irrigation treatment, water-urea treatment and water-compound fertilizer treatment in non-rhizosphere soil respectively. CKRS, HRS, NRS and FRS represent the control group, irrigation treatment, water-urea treatment and water-compound fertilizer treatment in the rhizosphere soil, respectively. Enzyme Commission (EC) Numbers can be inquired in the website: https://www.brenda-enzymes.org/all_enzymes.php.

**References**

1. Li KY, Zhao YY, Yuan XL, Zhao HB, Wang ZH, Li SX, Malhi SS. 2012. Comparison of Factors Affecting Soil Nitrate Nitrogen and Ammonium Nitrogen Extraction. Communications in Soil Science & Plant Analysis 43:571-588.

2. You Y, Chu S, Chi Y, Chen X, Wang J, Hayat K, Yang X, Müller C, Zhang D, Zhou P. 2021. How bacteria remediate soil nitrate for sustainable crop production. Journal of Cleaner Production 328.

3. You Y, Chi Y, Chen X, Wang J, Wang R, Li R, Chu S, Yang X, Zhang D, Zhou P. 2022. A sustainable approach for bioremediation of secondary salinized soils: Studying remediation efficiency and soil nitrate transformation by bioaugmentation. Chemosphere 300.

4. Glavas N, Smuc NR, Dolenec M, Kovac N. 2015. The seasonal heavy metal signature and variations in the microbial mat (petola) of the Seovlje Salina (northern Adriatic). Journal of soil & sediments 15:2359-2368.
